# A tide prediction and tide height control system for laboratory mesocosms

**DOI:** 10.7717/peerj.1442

**Published:** 2015-11-24

**Authors:** Luke P. Miller, Jeremy D. Long

**Affiliations:** 1 Hopkins Marine Station, Stanford University, Pacific Grove, CA, United States; 2 Department of Biological Sciences, San Jose State University, San Jose, CA, United States; 3 Coastal and Marine Institute Laboratory, San Diego State University, San Diego, CA, United States

**Keywords:** Intertidal, Estuary, Shore height, Mesocosm, *Spartina foliosa*

## Abstract

Experimental mesocosm studies of rocky shore and estuarine intertidal systems may benefit from the application of natural tide cycles to better replicate variation in immersion time, water depth, and attendant fluctuations in abiotic and edaphic conditions. Here we describe a stand-alone microcontroller tide prediction open-source software program, coupled with a mechanical tidal elevation control system, which allows continuous adjustment of aquarium water depths in synchrony with local tide cycles. We used this system to monitor the growth of *Spartina foliosa* marsh cordgrass and scale insect herbivores at three simulated shore elevations in laboratory mesocosms. Plant growth decreased with increasing shore elevation, while scale insect population growth on the plants was not strongly affected by immersion time. This system shows promise for a range of laboratory mesocosm studies where natural tide cycling could impact organism performance or behavior, while the tide prediction system could additionally be utilized in field experiments where treatments need to be applied at certain stages of the tide cycle.

## Introduction

Because of the abiotic and biotic heterogeneity found in intertidal communities, controlled laboratory mesocosm experiments often provide important insight into how these communities function (Dethier, Williams & Freeman, 2005; Matassa & Trussell, 2011; Stachowicz et al., 2008). Traditional methods of manipulating water level in these mesocosms ranged from completely submerging intertidal organisms to creating tides with fixed submergence depths and rapid tidal changes. Given that tidal conditions (e.g., frequency and length of emersion) drive abiotic conditions in the intertidal zone (Denny et al., 2009), and influence the behavior, performance, and survival of intertidal seaweeds, plants, and invertebrates, there is a pressing need to develop mesoscosm systems that create more ecologically realistic tidal conditions.

The ebb and flood of tides strongly affect intertidal organisms. For example, the timing of gamete release by seaweeds can be determined by the tides (Pearson & Brawley, 1996). For intertidal plants, inundation determines growth (Bonin & Zedler, 2008; Padgett & Brown, 1999; Ravit et al., 2007), oxygen conductance (Maricle & Lee, 2007), and survivorship (Woo & Takekawa, 2012). Tidal conditions can also affect animals, including their habitat selection (Davies, Edwards & Williams, 2006; Koch, 1989; Richardson, Ibarrola & Ingham, 1993), parasite release (Curtis, 1993), egg hatching (Kellmeyer & Salmon, 2001), feeding rates (Charles & Newell, 1997; Noël et al., 2009), and movement (Ng & Williams, 2006; Silva et al., 2010; Williams & Morritt, 1995). Thus, a broad array of organisms is impacted by tidal conditions.

Traditional methods of manipulating water level in aquarium tanks have included simple high to low transitions created by removing a drain standpipe to drop the water level, by opening a low-mounted drain valve to drain the tank, or by turning on electric pumps to flood the tank during high tide. These systems often run on a simple fixed daily cycle (i.e., 12 h high, 12 h low), or they may be timed to only submerge soil and plants for a few hours during the day (Adams & Bate, 1995; Boorman, Hazelden & Boorman, 2001; Paganini, Miller & Stillman, 2014). Water levels are normally changed in a binary fashion, with a quick transition from high to low set by the flow rate of the drain pipe. In some cases the transition from high to low water may be prolonged by actively pumping water from the tank at intervals, or carefully sizing the drain pipe to slow the outflow of water (Li et al., 2011; Woo & Takekawa, 2012). In a small number of studies, the timing of tidal inundation has been designed to also recreate the natural progression of high and low tide times from day to day, with each maximum or minimum coming later than the preceding day’s tide (Li et al., 2011; Miller, Matassa & Trussell, 2014; Pincebourde, Sanford & Helmuth, 2009). Studies using alternating water levels with estuarine systems have explored the role of tidal submersion on soil salinity, soil water potential, drainage, and the resulting effects on plant growth rates (Adams & Bate, 1995; Boorman, Hazelden & Boorman, 2001; Li et al., 2011; Woo & Takekawa, 2012). With rocky intertidal systems, these systems have been employed to recreate temperature and desiccation stresses at low tide that could impact animal performance (feeding rate, metabolic rate, and growth rate) under present (Pincebourde, Sanford & Helmuth, 2009) or future climate conditions (Miller, Matassa & Trussell, 2014) and in concert with additional variables such as water pH (Paganini, Miller & Stillman, 2014).

We implemented a tide height control system (THC hereafter) for an aquarium system using a microcontroller to calculate the predicted tide height and control a motor-driven drain height adjustment system for continuous depth control. Our goal was to smoothly vary water depth in deep containers holding estuary plants potted in natural soil, creating high and low tide conditions that followed the natural progression of tide heights and timing from day to day while creating drainage in the potted soil similar to what occurs in natural soil in the field. In addition, the goal was to produce a low-cost system that could operate beyond the reach of a persistent network connection and does not require a full laptop or computer to run. Using the THC system, we were able to match the rise and fall of the mixed semidiurnal tides at our nearby field site within our laboratory containers, including accounting for the natural 24 h 50 min lunar tidal day length that shifts each high and low tide later on each subsequent day. We used this system to examine the effects of tidal submersion duration at different shore heights on the growth rate of the cordgrass *Spartina foliosa* infested with scale insects.

## Methods

Tide height predictions for the THC were generated continuously using a stand-alone microcontroller (Arduino Uno, http://arduino.cc) with attached real-time clock that stored the date and time in local standard time. The microcontroller was programmed to make tide height predictions for the current date and time for the local National Atmospheric and Oceanic Administration (NOAA) tide reference station (San Diego Bay, San Diego, California, USA, mean tide range 1.75 m) using tidal harmonic data generated by the NOAA tide station and extracted from the open-source XTide tide prediction application (http://flaterco.com). NOAA tide monitoring stations measure water levels over many years and use these data to generate a set of 37 or more tidal harmonic constituent values that account for the influence of celestial cycles and local topographic features (Hicks & Szabados, 2006). By combining the specific harmonic constants for a tidal reference station with a date and time (used to estimate position of the moon and sun), it is possible to generate current (and past or future) tide height predictions relative to the zero tide datum (Mean Lower Low Water, MLLW). We provide an online archive for the R code (R Development Core Team, 2015) used to extract the relevant site data and to generate the Arduino code (C + + code) that runs the Arduino tide prediction routine, along with data for 140 NOAA sites around the mainland US, Hawaii, Alaska, and the Caribbean.

**Figure 1:**
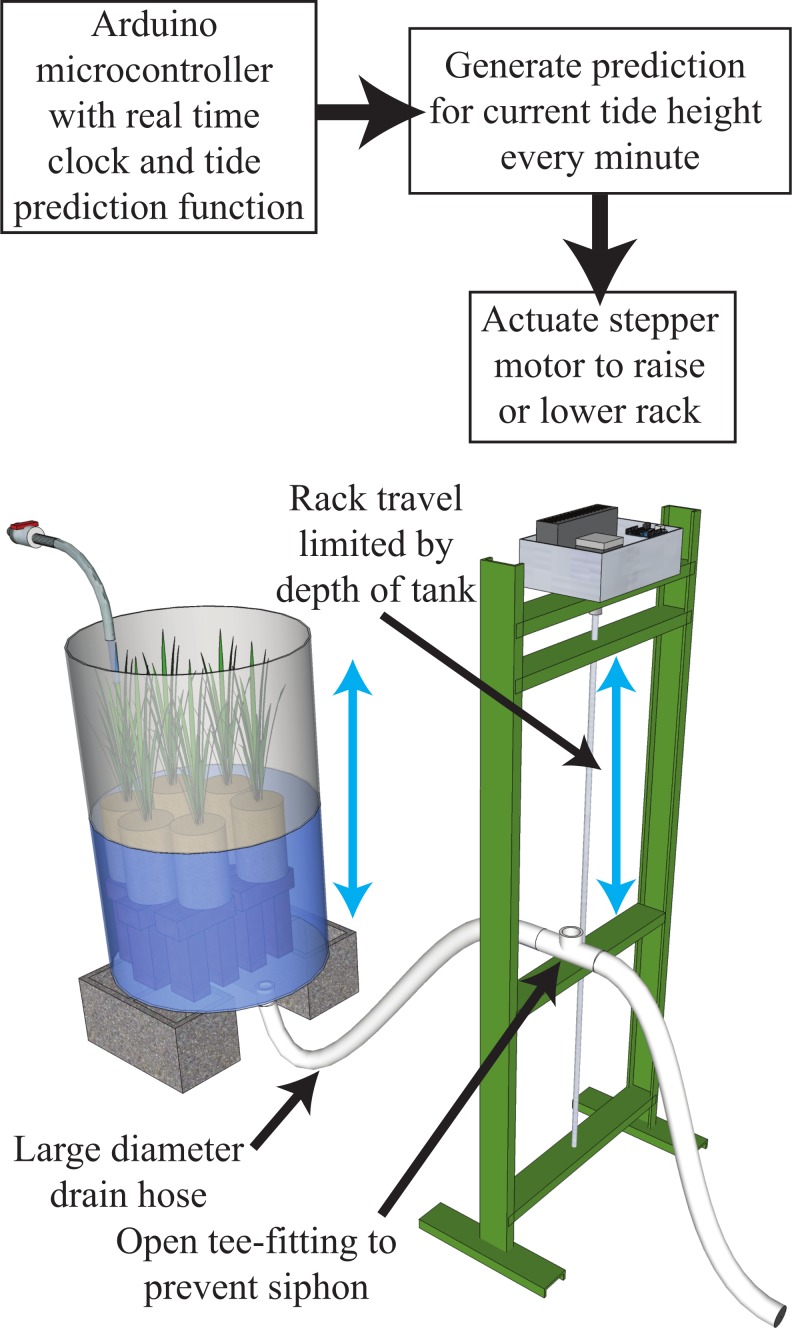
Functional diagram and illustration of the tide height control system. The microcontroller system is contained within the box at the top of the rack. A motor contained in the box drives the central lead screw of the rack up or down in time with the tide, raising or lowering the traveling rack carriage to change the height of the drain for the aquarium tank (88 cm tank height in this experiment). The water level in the tank will naturally equalize with the height of the traveling carriage. Multiple tanks can be controlled simultaneously by running each drain hose to the traveling carriage of the rack, and the simulated shore height of the tanks can be changed by adjusting the height of an individual tank relative to the rack, while the rack can also be programmed to travel within different ranges of the tide excursion.

We used the microcontroller to predict the current tide height every minute, and then actuated a motor-driven rack to raise and lower the drain height of several tanks (Fig. 1). The microcontroller interfaced with a stepper motor via a stepper motor controller (see online archive for specific parts lists). The stepper motor drove an acme-thread lead screw (Roton, Kirkwood, Missouri, USA), which moved a traveling nut 0.1 inches per rotation of the lead screw. The controller, motor, and lead screw were mounted in a rack standing 1.2 m tall. A “carriage” was mounted to the traveling nut on the lead screw, allowing the entire carriage to be driven up and down in the rack frame via the lead screw.

The aquaria for this experiment were 200 L plastic barrels (88 cm height, 59 cm diameter), with a 3.8 cm bulkhead fit in the bottom to serve as a drain. PVC pipe (3.8 cm) ran from the barrels to the base of the THC rack, where a section of large-diameter hose (1.6 cm ID) connected to the moving carriage on the tide rack. At the carriage, the hose was attached to a PVC tee fitting with the third leg open and oriented straight up. An additional section of large-diameter hose was attached to the opposite leg of the tee fitting and ran to a floor drain. The open leg of the tee fitting prevented the formation of a siphon during operation.

The water level in each barrel tracked the height of the rack carriage, so that as the carriage descended, the height of the water in each barrel attempted to equalize with the height of the carriage, causing the water level in the barrel to drop. The incremental shift in height of the carriage from minute to minute resulted in a very small head pressure on the drain hose. If small diameter drain hose was used, adequate drainage would be prevented by the wall drag and surface tension effects until a sufficient head pressure existed in the barrel to overcome that drag. Large diameter hose (1.6 cm) minimized this drag, allowing drainage to proceed even at small water height differentials between the barrel and carriage. During an ascending tide, the barrel water level would fill and rise to meet the new higher position of the carriage on the tide rack until the two heights equalized.

We used this system to examine the influence of shore elevation on *Spartina foliosa* (Pacific cordgrass). Elevation has previously been shown to strongly affect growth of a congener (*S. alterniflora*) in a field transplant experiment (Bertness, 1991). We collected *S. foliosa* and soil from Sweetwater Marsh (San Diego, CA) and brought them to San Diego State University’s Coastal and Marine Institute Laboratory. All plants were infested with herbivorous scale insects, *Haliaspis spartina*. Because scale insect density was variable at the time of collection, we counted the density of *H. spartina* on each plant, assigned them to one of five rankings of density, randomly selected one plant from each ranking, and placed plants into replicate barrels. We compared the growth of *S. foliosa* in mesocosms at three shore elevations (2.0, 1.8, and 1.6 m above MLLW) which would be submerged for different lengths of time depending on the tidal cycle. These elevations represent the upper end of *S. foliosa*’s elevational range—the highest densities of *S. foliosa* are typically found ∼1.0 m above MLLW. We chose the higher elevations because these areas typically have the highest densities of scale insects.

Three replicate barrels were positioned at each elevation, with each barrel containing five potted *S. foliosa*. Barrels at different elevations were randomly interspersed amongst each other. The plants were potted in 2.8 L pots (depth = 17 cm) filled with estuary soil. The pots were elevated inside each barrel so that the surface of the soil in the pot was at a height that would leave it submerged during predicted tide heights above the chosen shore elevation. As the tide dropped, the plant stems and soil would gradually be exposed, and water was able to drain from the soil in each pot. The three shore elevations were established by adjusting the height of the barrels relative to the tide rack.

**Figure 2:**
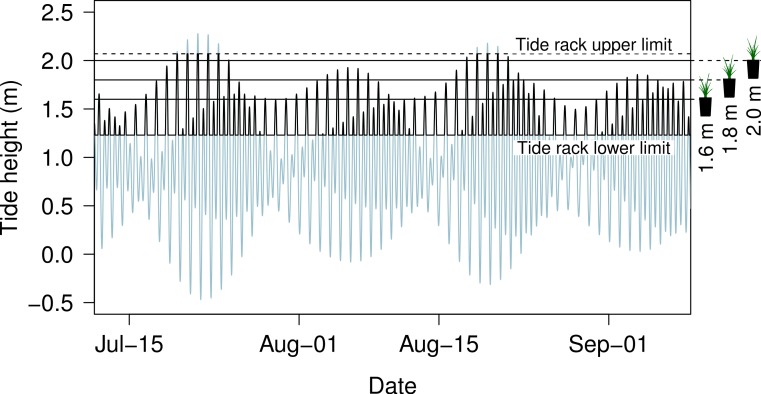
Predicted tide cycles for San Diego Bay during the 60 day experiment from July 11 to September 9 2013. The dark region represents the tide range that the tide control rack was capable of traveling through. Water height was held static at the upper or lower limits of the dark region when the natural tide exceeded the travel limits of the tide rack. The heights of the *Spartina* pots are shown at the right.

The upper travel limit of the THC carriage was defined as 2.07 m above MLLW. The total travel of the rack was limited to 0.84 m, so that the lower travel limit was at a tide height of 1.23 m above MLLW. When the natural tide cycle exceeded the lower (or upper) limit of the THC travel, the carriage halted at that limit until the tide was predicted to rise (or fall) above (or below) that limit again. During these time periods, the water level in the barrel remained equal to the carriage height. During a pilot experiment, we measured the water height difference at the PVC tee on the tide rack and water level in a barrel over a tide range of 0.21 m to ensure that the water level was following the rack movement within the travel range of the THC system. The exact height of the upper limit of the rack could be specified in software on the microcontroller, allowing the re-creation of different tidal ranges depending on the needs of the experiment. The experiment was run for 60 d from July 11 to September 9 2013, during which the maximum predicted tide height was 2.27 m and the minimum tide height was −0.42 m, though the tidal excursion in the barrels was limited to the values given above (Fig. 2). The plant height, measured as the distance from the soil surface to the tip of the longest leaf of each *S. foliosa*, was measured at the start of the experiment and every 7–9 days subsequently.

Because there were no differences among elevation treatments in the length of the longest leaf at the start of the experiment (*F*_2,51_ = 0.85, *p* = 0.44), we measured plant growth as the difference between the maximum leaf length and starting leaf length. Additionally, because we did not observe an effect of starting insect density on plant growth, we pooled all plants within each barrel to account for the nesting of five plants in each barrel (thus, we had a single growth value for each of our three replicate barrels for each elevation). Upon examination of the data, we observed heterogeneous variances between our elevation treatments and so analyzed our data with a Kruskal–Wallis test.

We also calculated scale insect population growth by dividing the maximum number of scales per stem by the starting number of scales per stem. This provided us with insight into the performance of scales at each elevation. Although scale dispersal primarily occurs on the natal plant (J Long, pers. obs., 2012), we pooled all plants within each barrel to account for the lack of independence. As with plant growth, we analyzed the data with a Kruskal–Wallis test. Analyses were carried with R 3.1.3.

## Results

Predictions from the THC tide calculation algorithm matched predictions from NOAA predictions (http://tidesandcurrents.noaa.gov/) within 1 cm at all times. Water levels in the aquaria barrels followed the height of the tide rack within 1–2 cm (1.25 ± 0.4 cm, mean ± 1 SE), so that realized tide heights in the barrels followed the predicted rise and fall of the tides, within the travel limits of the tide rack. The upper limit of the tide rack travel (2.07 m) was set slightly above the soil surface of the highest elevation treatments in our mesocosm experiment. The soil in the two lower elevation treatments was completely inundated on 49 occasions (tides above 1.6 m elevation) and 23 occasions (tides above 1.8 m elevation) during the 60 day experiment, while the soil in the highest elevation treatment was only covered on 11 days (Table 1).

**Table 1:** Summary of the number and duration of events where the soil surface of plant pots was submerged by a high tide event. The experiment ran for 60 days (1440 h).

Shore height (m)	Number of days with soil surface submergence event	Total hours with soil surface submerged	Percentage of time with soil surface submerged
1.6	49	161	11.2
1.8	23	58	4.1
2.0	11	22	1.5

**Figure 3:**
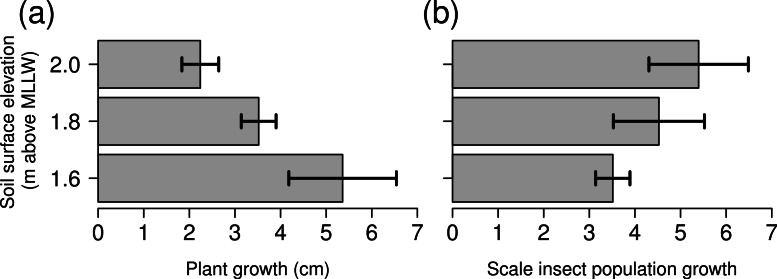
Growth of *Spartina foliosa* plants (A) and proportional growth of scale insect populations (B) in mesocosms connected to the tide controller at three elevations. Values are mean ± 1 SE, *n* = 3 replicate barrels per tide height.

The growth of *Spartina foliosa* depended upon tidal height, with lower plants growing more than twice the amount of higher plants (Fig. 3A, Kruskal–Wallis test, *H* = 6.489, *p* = 0.039). In contrast, the population growth rates of scale insects did not depend upon tidal height (Fig. 3B, Kruskal–Wallis test, *H* = 2.222, *p* = 0.329).

## Discussion

The ability to recreate natural cycling of tide height and timing allows researchers to better control what may be an important aspect of laboratory mesocosm experiments for estuary or rocky shore systems. We present a simple, standalone microcontroller system that generates real tide predictions, without the need for an external network interface, and that will automatically resume function following loss of power. Because the THC system’s real time clock carries a backup battery to retain the date and time, an interruption of power will result in the system rebooting and returning to the current predicted tide height when mains power is restored. These predictions are coupled with a novel motor-driven rack system to continuously manipulate the standing height of water in an aquarium, allowing smooth changes in tide levels.

We used the THC system to observe the effects of different tidal inundation durations on *Spartina foliosa*, a native cordgrass in marshes on the west coast of North America. Members of the genus *Spartina* have differing tolerances for the amount of time they spend submerged, and many estuaries exhibit a marked zonation in marsh plants with increasing tide height. The transition between different marsh plant species across shore elevation gradients is driven in part by competition and herbivory, but can also be a result of edaphic conditions influenced by the tide (Bertness, 1991). In this mesocosm experiment, reduced leaf elongation was associated with the prolonged aerial emersion produced by our higher shore elevation treatments.

In this experiment we simulated the tide cycling at a high shore height (1.23–2.07 m above MLLW), leaving the *S. foliosa* exposed for much of the time. The THC system can easily be adapted to simulate lower-shore submersion and emersion regimes by changing the reference shore height in the controller software. For example, if the goal was to recreate conditions in the 0–0.84 m shore height range (or any other range), changing a single value in the software could set the rack upper limit to be 0.84 m above MLLW. In this scenario, the THC rack would then alter the water level in the barrel whenever the predicted tide moved between 0.84 and 0 m, and the barrel would remain full whenever the predicted tide was above 0.84 m. If the researcher desired a larger range of movement, then deeper aquaria and a taller THC rack could be built, and only two values in software (upper limit and available rack travel) would need to be adjusted. The motor driven rack in this experiment was set up to drain nine separate aquaria, and additional aquaria could be included by modifying the system to use a more powerful motor to lift the weight of additional drain hoses.

Outside of the application described here for controlling water height in an estuarine mesocosm experiment, the THC tide prediction function could be used in a number of other scenarios. The system can be adapted to control aquarium flow in simpler high/low tide regimes that may still require a natural progression of the timing of high and low tide from day to day. The microcontroller tide calculations described here have been used to actuate an electric drain valve mounted on the bottom of a tank, so that a closed valve forces the water level to rise and exit via a high mounted drain at high tide, while an open valve causes the water level to drop to simulate low tide. Alternatively, the controller has been used to regulate the delivery of water spray so that it only occurs during high tide periods, simulating the periodic inundation created by wave action. This implementation has been used to successfully maintain rocky intertidal grazers such as high shore *Lottia* limpets that dislike being permanently submerged (Miller et al., 2015). Finally, the small size and lower power consumption of the microcontroller system allow it to be used in field locations where it can be powered by battery or solar cells. Lee et al. (D Lee, pers. comm., 2015, Virginia Commonwealth University) used the tide predictions generated by this system to control the timing of water release in an estuarine field experiment aimed at manipulating the duration of soil surface wetting to imitate the effects of higher sea level. In a scenario such as this, where manipulations need to be timed to coincide with the constantly shifting tide cycles in order to conserve power or supplies, our system can allow accurate timing and will run for long periods of time on minimal power. The Arduino microcontroller making the tide predictions consumed about 20 mA in the original configuration (not including the motor driven rack), but this power draw could be reduced to 0.3 mA or less with alternate Arduino-compatible microcontroller hardware and software changes. The microcontroller could also be easily modified to track and manipulate other environmental variables in addition to tide height, such as temperature or light, and to serve as a datalogger, using inexpensive commercially-available modules developed by the open source community.

The software libraries provided in the online materials are presently limited to sites for which NOAA freely provides data under its mandate that United States government products be made available to non-commercial and commercial users. Many other countries make their tide harmonic data available only as a commercial product, but if the user can gain access to the tidal harmonic constituents for their site elsewhere in the world, the underlying open-source tide prediction software used here can be updated to substitute those values, as described in the Arduino C + + source code provided in the online archive.

## Additional Information and Declarations

### Data Availability

The following information was supplied regarding data availability: The software and hardware designs described in this manuscript have been made freely available in a permanent digital archive at Stanford University, http://purl.stanford.edu/rb471wt3944. Github.com: https://github.com/millerlp/Tide_calculator and https://github.com/millerlp/Tide_controller.
